# Neuropsychiatric outcomes before and after switching to dolutegravir-based therapy in an acute HIV cohort

**DOI:** 10.1186/s12981-019-0257-8

**Published:** 2020-01-07

**Authors:** Phillip Chan, Orlanda Goh, Eugène Kroon, Donn Colby, Carlo Sacdalan, Suteeraporn Pinyakorn, Peeriya Prueksakaew, Peter Reiss, Jintanat Ananworanich, Victor Valcour, Serena Spudich, Robert Paul, the RV254/SEARCH 010 Research Team

**Affiliations:** 1 SEARCH, The Thai Red Cross AIDS Research Centre, Bangkok, Thailand; 2 The Henry M. Jackson Foundation for the Advancement of Military Medicine, Bethesda, MD USA; 3 United States Military HIV Research Program, Walter Reed Army Institute of Research, Silver Spring, MD USA; 4 Department of Global Health, Amsterdam University Medical Centers, University of Amsterdam, and Amsterdam Institute for Global Health and Development, Amsterdam, The Netherlands; 5 Memory and Aging Center, Department of Neurology, University of California San Francisco, San Francisco, CA USA; 6 Center for Neuroepidemiology and Clinical Neurological Research, Yale University, New Haven, CT USA; 7 Missouri Institute of Mental Health, University of Missouri-St. Louis, St. Louis, MO USA

**Keywords:** Dolutegravir, Neuropsychiatric adverse events, Depression, RV254, Cognitive performance

## Abstract

**Introduction:**

Dolutegravir (DTG)-based antiretroviral therapy (ART) is currently the first-line treatment for people living with HIV. Neuropsychiatric adverse events (NP-AEs) have been reported with DTG but neuropsychiatric symptoms have not been systemically quantified using structured scales. This study examined mood and cognitive parameters before and after a planned transition from non-DTG to DTG-based ART within a longitudinal study of acute HIV infection (AHI).

**Methods:**

RV254 AHI cohort participants on ≥ 24 weeks of ART initiated at AHI underwent sequential assessments before and after the switch including: (1) Patient Health Questionnaire-9 (PHQ-9), a 9-item survey (scores 0–27) that evaluates somatic and affective/cognitive symptoms of depression; (2) a 2-Questions screening that has been validated locally for depression; (3) Distress Thermometer (scores 0–10); and 4) administration of a 4-test neurocognitive battery sensitive to HIV.

**Results:**

254 individuals (95% male, median age 30) switched to a DTG-based regimen after a median 144 weeks of ART. Serial assessments were completed at a median of 19 weeks before and 37 weeks after DTG. There was a modest but statistically significant increase in PHQ-9 scores after DTG (pre-switch: 5 [IQR 1–7] vs. Post-switch: 5 [IQR 2–8], p = 0.009). The percentage of participants with at least moderate depression (PHQ-9 ≥ 10) increased from 10 to 16% (p = 0.006), but the frequency of moderate-severe depression (PHQ-9 ≥ 15) remained unchanged (3%). No volunteer reported NP-AEs within the study period. Somatic symptoms of depression increased more than cognitive/affective symptoms. Plasma viral suppression (HIV-1 RNA < 50; p = 0.005) and PHQ-9 ≥ 10 (p < 0.001) before switch were linked to lower PHQ-9 scores after DTG in multivariable analysis. Performance on all neuropsychological tests, except grooved pegboard test, improved modestly after DTG (all p < 0.05).

**Conclusion:**

After a median duration of 37 weeks of DTG use, there was a modest increase in the higher quartile of PHQ-9. This increase was associated with a rise in moderate depression symptoms but not the more severe forms of depression on PHQ-9. No clinically relevant NP-AEs were reported. Pre-existing depression was not associated with subsequent worsening of symptoms after DTG. Cognitive test performance improved post-DTG but could be due to practice effect.

## Introduction

Dolutegravir (DTG) is a potent second-generation HIV integrase strand inhibitor with a high genetic barrier to resistance and favourable tolerability [1]. The World Health Organization recently recommended the use of DTG-based regimens as first-line antiretroviral therapy (ART) for people living with HIV (PLWH). However, clinical reports have raised concern regarding the risk of DTG-associated neuropsychiatric adverse events (NP-AEs) [2–4]. The rate of DTG discontinuation for NP-AEs ranged from 1 to 6% in previous studies [2–4].

Prior studies including self-reported NP-AEs described increased rates of insomnia in a minority of individuals after starting a DTG-based regimen. Increased depression has also been reported, particularly among individuals with a history of depression before DTG initiation [3]. However, the impact of DTG-based ART on the dimensional characterization of depression has not been explored. Additionally, neurocognitive performance before and after DTG-based ART is not well defined [2]. We previously reported that DTG was well tolerated with few discontinuations among young men who switched from a non-DTG to a DTG-based regimen [5]. This follow-up report focuses on affective and somatic dimensions of depression, and cognitive performance before and after switch to DTG.

## Methods

### Study design

**Fig. 1:**
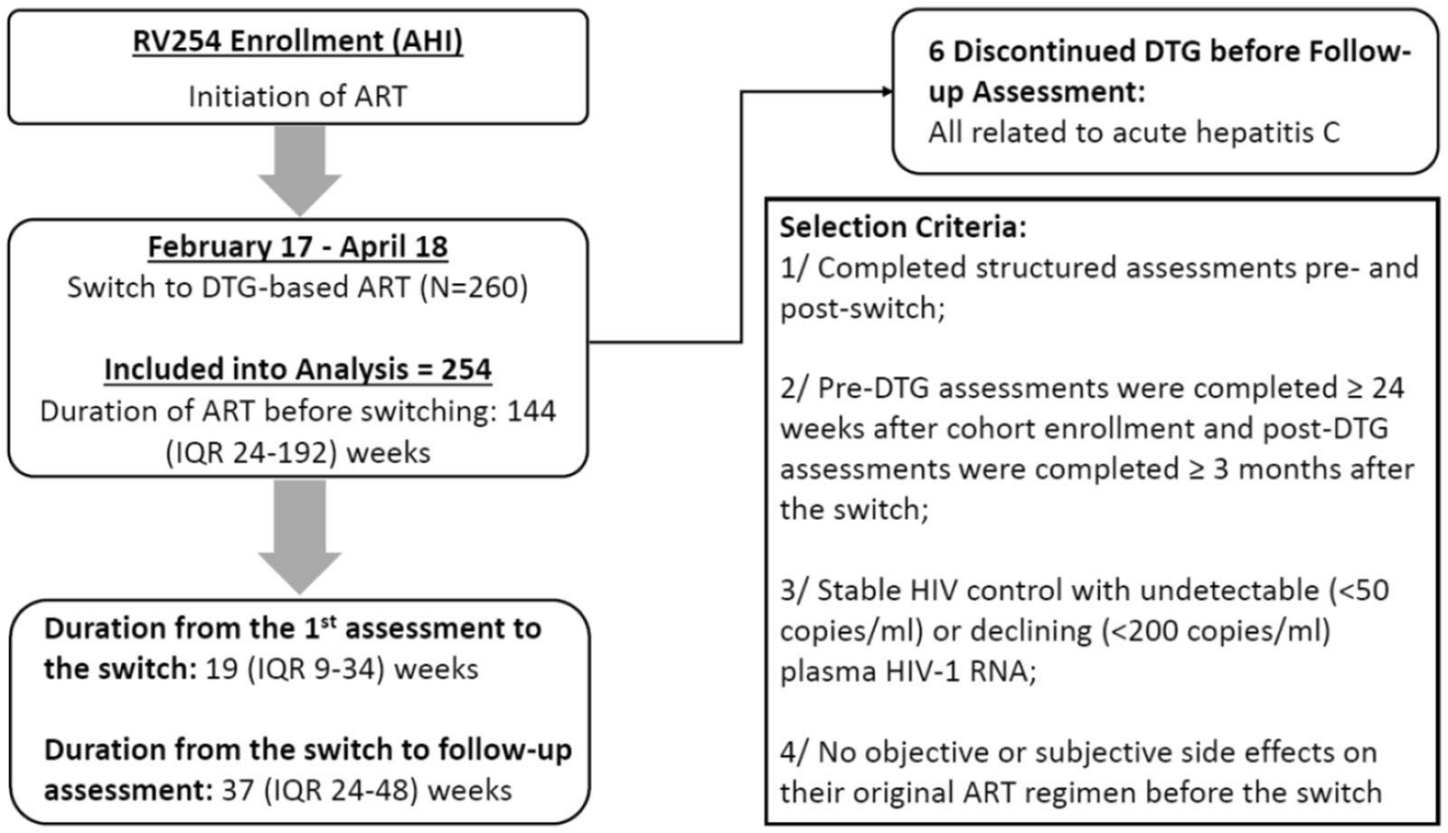
Study design and selection criteria. *AHI* acute HIV infection, *DTG* Dolutegravir, *ART* antiretroviral therapy

We examined prospective data from the SEARCH010/RV254 cohort, an ongoing study of long-term outcomes following ART initiation during acute HIV infection (AHI) started in April 2009 (NCT00796146 and NCT00796263) [6]. Almost all participants initiated Efavirenz (EFV)-based ART within days (median = 0; [IQR: 0–1]) after AHI diagnosis. They underwent regular clinical follow-up, laboratory blood tests, neurocognitive assessment and self-reported mood symptoms questionnaires (see below). The study protocol was approved by the institutional review boards of all relevant collaborating institutions. All participants provided written informed consent. Starting in March 2017, cohort participants systematically switched to a DTG-based regimen (Fig. 1). Participants with elevated liver enzymes (grade III or above) or unstable liver disease were excluded from switching.

### Participants selection

Participants who fulfilled the following criteria by 30th April 2018 were included for analysis: 1/completed structured assessments of mood, neurocognitive assessment and HIV-related laboratory tests (plasma HIV-1 RNA, CD4+ and CD8+ T cell levels) before and after the switch; 2/pre-DTG assessments were completed at least 24 weeks after cohort enrolment (AHI) and post-DTG assessments were completed at least 3 months after the switch; 3/stable virologic control with undetectable (< 50 copies/ml) or declining (< 200 copies/ml) plasma HIV-1 RNA; and 4/without objective or subjective side effects on their pre-DTG ART regimen. The second and third criteria aimed to prevent impact from depression and anxiety associated with AHI diagnosis and the biological effect driven by plasma viremia [7], whereas the last criterion aimed to prevent cognitive and psychological benefits gained from switching from an ART regimen with known side effects.

### Neuropsychiatric assessment

Mood assessments included the Patient Health Questionnaire-9 (PHQ-9), 2Q-Depression screen and Distress Thermometer (DT), which have been validated for use in Thailand [8–11]. The PHQ-9 is a 9-item survey (score range 0–27) derived from DSM-IV criteria for depression [12]. It can be further categorized into somatic (sleep/appetite/energy level, questions 3–5) and affective/cognitive (questions 1, 2, 6–9) components of depression. PHQ-9 total scores ≥ 10 and ≥ 15 have been used to detect moderate and moderate-severe depression, respectively [12]. The 2Q-Depression screen was developed and validated by the Thai Ministry of Public Health to serve as a rapid assessment of clinically relevant depression [8]. The 2Q-Depression screen asks participants two yes/no questions related to sadness and loss of interest or pleasure in daily activities [8]. The DT is a self-report measure of emotional stress that utilizes an image of a thermometer to guide severity ratings of stress and anxiety from 0–10 [10, 11].

### Neurocognitive assessment

Neurocognitive tests included measures of fine motor speed and dexterity (non-dominant hand Grooved Pegboard test (GPB; Lafayette Instrument Company, Lafayette, USA), psychomotor speed (Color Trails 1 and Trail Making A; PAR, Inc., Lutz, USA) and executive functioning/set shifting (Color Trails 2; PAR, Inc., Lutz, USA; see [13] for complete information). In the parent study cohort (RV254), participants are regularly assessed by this battery longitudinally, since enrolment at pre-treated AHI. As all the selected participants had to be followed for more than 24 weeks after enrolment (2nd criterion), they would have completed the neurocognitive test battery on at least three occasions (baseline, week 12 and 24) prior to the DTG switch. This design consideration minimized the potential confound of practice effect before and after the switch, which is most obvious between the first and second assessment [14]. Raw scores were standardized to Thai normative data [13] and z-scores for each test were averaged to provide a measure of overall neuropsychological performance (NPZ-4).

### Data analysis

Results were reported as median and interquartile range (IQR) or frequency and percentage, as appropriate. Plasma viral suppression was defined as HIV-1 RNA < 50 copies/ml. McNemar and Wilcoxon signed-rank tests were used, as appropriate, to compare the outcomes before and after DTG. Multivariate linear regression examined factors that were correlated with the change in PHQ-9 scores between the 1st and 2nd assessment. Statistical analyses were performed using SPSS Version 18.0 (IBM Corp., Armonk, NY).

## Results

At the time of analysis, 260 participants who had switched to a DTG-based regimen fulfilled the selection criteria. Of note, 6 participants preferred not to switch to a DTG-based regimen due to pill burden (n = 5) or for unknown reason (n = 1). Six participants discontinued DTG before post-DTG assessment due to acute hepatitis C-related elevated liver enzymes; these individuals were excluded. No participants discontinued DTG because of subjective or elicited NP-AEs within the analysis period. Among the 254 participants included in this study, nearly all were Thai (99%) and male (95%), with a median age of 30 [IQR 25–36]. Participants switched to dolutegravir/abacavir/lamivudine (85%) or dolutegravir/tenofovir disoproxil fumarate/lamivudine in case of a positive HLA-B*5701 assay or chronic hepatitis B infection. The median duration of ART prior to the planned switch was 144 [IQR 24–192] weeks; 82% were previously on EFV-based ART, 13% were on a boosted protease inhibitor (mostly lopinavir)-based ART, 5% were on rilpivirine-based ART, and one individual was on a raltegravir-based regimen.

The median duration from pre-switch assessment to the switch to DTG was 19 [IQR 9–34] weeks, and from the switch to the follow-up assessment was 37 [IQR 24–48] weeks. Table 1 shows all the tested parameters before and after the switch. At follow-up, the frequency of plasma viral suppression increased from 96 to 98% (p = 0.070). Additionally, CD4+ T-cell count (pre-switch: 624 [IQR 512–783] vs. post-switch: 662 [IQR 530–833], p < 0.001) and CD4/CD8 ratio (pre-switch: 1.09 [IQR 0.85–1.41] vs. post-switch: 1.12 [IQR 0.87–1.43], p = 0.026) were higher at follow-up.

**Table 1:** Parameters before and after transition to dolutegravir (N = 254)

	Pre-switch^d^	Post-switch^d^	p value
CD4+ T-cells (cells/μl)	624 (512–783)	662 (530–833)	< 0.001
CD8+ T-cells (cells/μl)	574 (449–787)	618 (482–797)	0.155
CD4/CD8	1.09 (0.85–1.41)	1.12 (0.87–1.43)	0.026
NPZ-4	0.70 (0.31–1.10)	0.88 (0.37–1.19)	< 0.001
Color Trails 1 z-score	1.15 (0.59–1.56)	1.30 (0.64–1.74)	0.001
Color Trails 2 z-score	0.61 (0.14–1.11)	0.86 (0.40–1.22)	< 0.001
Grooved pegboard test z-score	0.54 (− 0.20–1.05)	0.64 (− 0.09–1.10)	0.149
Trail making A z-score	0.75 (0.14–1.15)	0.80 (0.07–1.33)	0.037
PHQ-9 score	5 (1–7)	5 (2–8)	0.009
PHQ-9 ≥ 10, n (%)	24 (10)	40 (16)	0.006
PHQ-9 ≥ 15, n (%)	8 (3)	8 (3)	1.000
PHQ-9 somatic sub-score^a^	2 (0–3)	2 (1–3)	0.007
PHQ-9 cognitive/affective sub-score^b^	2 (0–4)	2 (0–5)	0.064
Major depression by 2Q-depression screening, n (%)	2 (1)	3 (1)	1.000
Distress thermometer score	2 (1–5)	2 (1–4)	0.898
Viral suppression, n (%)^c^	244 (96)	250 (98)	0.070

*NPZ-4* Composite z-score of the 4 neuropsychiatric tests, *PHQ-9* Patient Health Questionnaire-9

^a^Questions 3, 4, 5

^b^Questions 1, 2, 6, 7, 8, 9

^c^Defined as plasma HIV RNA < 50 copies/ml

^d^Median (IQR) is presented unless specified; Wilcoxon and McNemar test were used accordingly

### Mood symptoms before and after dolutegravir

Scores on the DT and the 2Q-Depression screen did not change after DTG (both p > 0.10). At follow-up, the total PHQ-9 score increased in 48% of participants, decreased in 31%, and remained unchanged in 21%, resulting in a modest but statistically significant increase in the PHQ-9 score after DTG, with an increase in the upper IQR (pre-switch: 5 [IQR 1–7] vs. post-switch: 5 [IQR 2–8], p = 0.009). The percentage of participants with at least moderate depression (PHQ-9 ≥ 10) increased from 10% (n = 24) to 16% (n = 40, p = 0.006), whereas the percentage of participants with moderate-severe depression (PHQ-9 ≥ 15) remained unchanged (3%).

Comparing the changes in the somatic and cognitive/affective subset scores of PHQ-9 showed a more prominent but modest change in the somatic sub-scores similar to that in the total PHQ-9 score. There were increases in somatic sub-scores (pre-switch: 2 [IQR 0–3] vs. post-switch: 2 [IQR 1–3], p = 0.007) and the cognitive/affective sub-scores (pre-switch: 2 [IQR 0–4] vs. post-switch: 2 [IQR 0–5], p = 0.064) at the lower and upper IQR respectively with unchanged median scores.

### Factors associated with PHQ-9 change after dolutegravir

Linear regression was employed to evaluate potential factors that were associated with the PHQ-9 change (i.e. PHQ-9 at 2nd assessment minus PHQ-9 at 1st assessment) (Table 2). In the univariate analyses, plasma viral suppression and PHQ-9 ≥ 10 before DTG were associated with lower PHQ-9 scores at follow-up (p = 0.003 and p < 0.001, respectively). Older age trended towards association with lower PHQ-9 scores after DTG (p = 0.096). CD4+ T-cell count and EFV use before DTG were not associated with subsequent change in PHQ-9 score. In the multivariate analysis, only pre-existing viral suppression (mean difference − 3.2, 95% CI [− 0.9 to − 5.4], p = 0.006) and PHQ-9 ≥ 10 pre-switch (mean difference − 2.7, 95% CI [− 1.2 to − 4.2], p < 0.001) remained independently associated with a decrease in PHQ-9 score.

**Table 2:** Factor correlation with PHQ-9 changes

	Univariable		Multivariable	
	PHQ-9 mean difference (95% CI)	p-value	PHQ-9 mean difference (95% CI)	p-value
Age	− 0.05 (− 0.01 to 0.008)	0.096		NS
Sex (male)	0.64 (− 1.4 to 2.70)	0.537		
CD4, every 100 cells/μl	− 0.03 (− 0.2 to 0.2)	0.713		
Viral suppression	− 3.4 (− 5.7 to − 1.2)	0.003	− 3.2 (− 0.9 to − 5.4)	0.006
PHQ-9 ≥ 10 before DTG	− 2.8 (− 4.3 to − 1.3)	< 0.001	− 2.7 (− 1.2 to − 4.2)	< 0.001
PHQ-9 ≥ 15 before DTG	− 6.2 (− 8.6 to − 3.7)	< 0.001		
EFV use before DTG	0.11 (− 1.1 to 1.3)	0.859		

PHQ-9 change = PHQ-9 at 2nd assessment minus PHQ-9 at 1st assessment

Statistical method: Linear regression with PHQ-9 change as dependent variable

Factors with p < 0.1 in the univariable model were included into multivariable analysis

*NS* not significant, *PHQ-9* Patient Health Questionnaire-9, *EFV* Efavirenz

### Neurocognitive tests performance

The mean neurocognitive performance, measured by the NPZ-4, increased modestly at follow-up (pre-switch: 0.70 [IQR 0.31–1.10] vs. post-switch: 0.88 [IQR 0.37–1.19], p < 0.001). Z-scores on the Color Trails 1 and 2 and Trail Making A were higher after DTG (all p < 0.05) while the performance on the GPB remained statistically similar (Table 1). Additional comparison between EFV (n = 207) and non-EFV users (n = 47) pre- and post-switch revealed statistically similar test performance (Table 3).

**Table 3:** Neuropsychological tests performance before and after DTG by EFV use at pre-switch

	EFV-based ART	Non-EFV-based ART	p-value	EFV-based ART	Non-EFV-based ART	p-value
NPZ-4	0.69 (0.32 to 1.10)	0.83 (0.27 to 1.14)	0.776	0.87 (0.37 to 1.19)	0.89 (0.31 to 1.21)	0.747
zCT1	1.13 (0.59 to 1.54)	1.25 (0.53 to 1.63)	0.667	1.29 (0.63 to 1.74)	1.36 (0.89 to 1.81)	0.689
zCT2	0.59 (0.12–1.10)	0.65 (0.26 to 1.15)	0.525	0.86 (0.40 to 1.20)	0.81 (0.17 to 1.33)	0.916
zGPB	0.55 (− 0.15 to 1.07)	0.44 (− 0.40 to 1.00)	0.379	0.66 (− 0.08 to 1.14)	0.56 (− 0.45 to 1.04)	0.857
zTrailA	0.75 (0.09 to 1.15)	0.79 (0.37 to 1.13)	0.902	0.84 (0.11 to 1.37)	0.65 (− 0.21 to 1.15)	0.126

Median (IQR) is presented

*ART* antiretroviral therapy; *EFV* Efavirenz, *NPZ-4* composite z-score of the 4 neuropsychiatric tests, *CT1* Color Trails 1, *CT2* Color Trails 2, *GPB* grooved pegboard test, *TrailA* trail making A

## Discussion

This study provides an integrated evaluation of mood symptoms and cognitive performance before and after a planned switch to DTG in individuals on stable ART initiated during AHI. All participants were maintained on stable ART before the switch with a median duration of 144 weeks. The current study leveraged an organized change in treatment regimen to a standardized DTG-based regimen among a large cohort who had almost universally achieved viral suppression using non-DTG ART. This is an important distinction to other studies that reported worsening of cognitive performance among those on DTG. All participants were stable on their pre-switch ART without objective or subjective side-effects, which helped to prevent additional benefit from switching to a DTG-based ART. After a median duration of 37 weeks of DTG, there were no discontinuations because of NP-AEs. The proportion of participants with moderate depression (PHQ-9 ≥ 10) increased after DTG, but the median total PHQ-9 score and the percentage of participants with moderate-severe depression (PHQ-9 ≥ 15) remained unchanged. Scores on the DT and the 2Q-Depression screen were similar after DTG. Taken together, our findings do not support a clear association between the use of DTG and worsening of clinically relevant mood symptoms in young male HIV-positive population.

The multivariable analysis aimed to identify potential contributors linked to PHQ-9 changes. It revealed that individuals who achieved viral suppression pre-switch were less likely to experience worsening of depression symptoms following DTG. Further, those with higher PHQ-9 scores pre-switch were not linked to higher scores at follow-up.

In subset analyses, both somatic and affective/cognitive dimensions of the PHQ-9 modestly worsened after DTG, but the magnitude of change was only statistically significant for the somatic subscale. Yagura et al. previously reported a potential link between supratherapeutic DTG levels and CNS side-effects [15], in which 88% of adverse events were somatic in nature (e.g. headache, dizziness, insomnia and restlessness). Elliot et al., however, reported no association between DTG pharmacokinetics (PK) and changes in sleep parameters or neurocognitive performance in PLWH aged 60 or older [4]. Additional work is needed to determine whether a subgroup of PLWH may be prone to DTG-related NP-AEs.

Modest improvements were observed in 3 out of 4 neurocognitive test scores and HIV clinical indices (CD4+ T-cell count and CD4/CD8 ratio). However, the improvement in neurocognitive test is unlikely driven by the switch to DTG. First, the pre-DTG z-scores in all 4 tests were within normal range and did not support any cognitive impairment among our participants pre-switch. Second, albeit multiple groups have reported a possible association between EFV use and worse cognitive performance [16–18] and the majority of our participants were on EFV pre-switch, pre-DTG cognitive test performances were similar between EFV and non-EFV users in our study. Thus, the improvement in test performance was less likely driven by elimination of any negative effects from EFV-related cognitive symptoms. Practice effect remains a plausible explanation [14], although the participants had already undergone repeated testing before the post-DTG assessment. The improvement in cognitive function may have also allowed greater insight into their bodily condition that led to the modest worsening of depression symptoms. However, as our participants were on average not cognitively impaired pre-switch and insight ability was not evaluated in the neurocognitive test battery, we are unable to examine this potential linkage. In any case, the observation that neurocognitive performance post-DTG was stable among this cohort of AHI is reassuring and is in line with previous work [4] that utilized different neurocognitive tests [19].

## Limitations

Our participants were mostly young males without additional complicating medical problems, who started ART during AHI and thus had few comorbidities and a relatively high CD4 nadir. Individuals with major psychiatric illness were not enrolled in the main protocol. These factors may restrict the generalizability of the findings to other settings, where PLWH of older age, with multiple co-morbidities, and/or more advanced immunosuppression are common. Our findings are also limited by the lack of a control group comprised of individuals who did not switch to DTG, which would allow a more robust assessment of DTG-related changes in mood and cognitive performance.

## Conclusion

Although there was a modest increase in the higher quartile of PHQ-9 after the switch, transition to a DTG-based ART is associated with relative stability in neuropsychiatric symptoms in a group of predominantly young male PLWH. The modest increase in somatic symptoms may explain the anecdotal reporting of increased neuropsychiatric side effects following use of DTG in large clinical trials. Additional studies are needed to inform outcomes among females, older PLWH, and individuals with chronic disease receiving a DTG-based regimen for HIV.

## Data Availability

The datasets used and/or analysed during the current study are available from the corresponding author on reasonable request.
